# UIDF-Net: Unsupervised Image Dehazing and Fusion Utilizing GAN and Encoder–Decoder

**DOI:** 10.3390/jimaging10070164

**Published:** 2024-07-11

**Authors:** Anxin Zhao, Liang Li, Shuai Liu

**Affiliations:** School of Communication and Information Engineering, Xi’an University of Science and Technology, Xi’an 710054, China

**Keywords:** image processing, single image dehazing, haze encoder, unsupervised learning

## Abstract

Haze weather deteriorates image quality, causing images to become blurry with reduced contrast. This makes object edges and features unclear, leading to lower detection accuracy and reliability. To enhance haze removal effectiveness, we propose an image dehazing and fusion network based on the encoder–decoder paradigm (UIDF-Net). This network leverages the Image Fusion Module (MDL-IFM) to fuse the features of dehazed images, producing clearer results. Additionally, to better extract haze information, we introduce a haze encoder (Mist-Encode) that effectively processes different frequency features of images, improving the model’s performance in image dehazing tasks. Experimental results demonstrate that the proposed model achieves superior dehazing performance compared to existing algorithms on outdoor datasets.

## 1. Introduction

With the rapid development of visual perception technology, imaging devices in industrial applications, such as intelligent security [1], autonomous driving [2], industrial manufacturing [3], and medical image analysis and diagnosis [4], can achieve automated tasks, including detection, classification, localization, recognition, and tracking. However, in outdoor environments, there are inevitably large amounts of suspended microscopic particles and gas mixtures, such as water vapor and smoke, in the air. Sunlight passing through these mixtures causes light scattering, resulting in haze [5]. Haze reduces image visibility, distorts colors, and diminishes distant scene details captured by imaging devices. This degradation impacts their performance in visual tasks like image classification and object detection. For instance, in mountainous areas, significant temperature differences between day and night can result in morning haze, preventing the comprehensive monitoring of power line statuses. Even re-imaging cannot avoid the impact of haze in real-time monitoring environments. Therefore, researching image dehazing technology is essential. Image dehazing [6] aims to eliminate or mitigate the haze effects caused by atmospheric scattering to improve image quality, enhance visual perception, and increase the performance and usability of images in various visual applications. Currently, researchers are enthusiastic about the study of single-image dehazing methods, broadly categorized into methods based on prior knowledge [7], methods based on the Retinex model [8], and deep learning-based image dehazing [9].

Typical image dehazing algorithms based on prior knowledge include dark channel prior [10], color restoration methods [11], natural scene statistics prior [12], and image enhancement techniques based on random field models [13]. However, these algorithms have significant limitations. For instance, the use of the dark channel prior algorithm in real haze removal scenarios can cause color distortion. It may mistakenly identify dark areas as haze, leading to artifacts or color shifts in dehazed images. This is particularly noticeable in high-contrast regions, resulting in blurred or lost image details, textures, and distant scene features, including edge information. Moreover, this algorithm assumes uniform consistency of atmospheric light in the image, whereas actual lighting conditions may vary, affecting the dehazing effect. Additionally, this algorithm is not suitable for outdoor sky-containing image dehazing tasks, as it may lead to halo artifacts in dehazed images. Nishino et al. [14] proposed a Bayesian dehazing model based on factorial Markov random fields, which jointly predicts scene reflectance and depth information. This method achieves some degree of dehazing effectiveness but often generates artifacts in regions close to infinite depth.

The core approach of image dehazing based on the Retinex model [15] involves decomposing the image into its illumination and reflection components. Next, the illumination component is estimated by convolving the image with a Gaussian function and the haze image. Then, the reflection component is obtained by dividing the original image by the estimated illumination component. Finally, the reflection component is enhanced to reconstruct the dehazed image. However, the Retinex model has certain limitations. It performs poorly when dealing with complex atmospheric conditions and varying light intensities, leading to the inaccurate separation of illumination and reflection components. Additionally, the model cannot adaptively adjust the scale of the Gaussian kernel based on the environment, resulting in poor color fidelity of the image. In recent years, deep learning has demonstrated powerful feature representation capabilities in image restoration tasks, prompting researchers to shift from traditional dehazing methods to deep learning-based approaches.

Deep learning-based image dehazing methods [16] can be broadly categorized into those based on physical models and those based on feature fusion learning methods. The former includes supervised learning methods like DehazeNet [17], AOD-Net [18], and DCPDN [19] and unsupervised learning methods like USID-Net [20], ZID [21], RefineDNet [22], and D4 [23]; the latter includes methods like EPDN [24], GFN [25], GridDehazeNet [26], among others. Cai et al. [17] proposed the DehazeNet dehazing network algorithm, which uses deep convolutional neural networks to estimate intermediate parameters of the atmospheric scattering model, establishing a mapping relationship between haze images and intermediate parameters. Boyi et al. [18] introduced the AOD-Net dehazing network, which utilizes deep convolutional neural networks for feature extraction to learn to estimate atmospheric light and transmission in haze images, achieving end-to-end training to restore input hazy images. Compared to traditional dehazing methods, deep learning-based dehazing methods have achieved superior performance. However, learning-based methods require the building of large paired datasets (matching high-definition and hazy images). Therefore, learning-based methods start with high-definition images, artificially setting different image degradation mechanisms to obtain matching hazy images. While superior in most tests compared to previous traditional methods, the generalization ability of these models is poor under real haze conditions due to dataset limitations.

To address the aforementioned issues, researchers have proposed unsupervised learning methods [27]. Engin et al. introduced the Cycle-Dehaze network [28] for image dehazing using non-aligned training set images. This network framework consists of two generators (G and F) and two discriminators (Dx and Dy). Generators G and F are used to approximate the process of hazy image inversion to clear images and clear image generation to hazy images, respectively. Discriminators Dx and Dy are used to assess the fitting capability of generators G and F. The algorithm enhances cycle consistency and perceptual loss to improve the quality of texture information recovery and generate visually better haze-free images. Zhao et al. proposed a weakly supervised RefineDNet [22], which combines dark channel prior and learning-based methods, using unpaired data for adversarial learning to improve the quality of dehazed images. Li et al. introduced an unsupervised USID-Net [20], which combines Cycle-Dehaze with OctEncoder to enhance the dehazing speed. Meanwhile, unsupervised learning addresses issues such as the need for paired datasets and poor generalization in supervised learning. However, unsupervised learning involves more complex network structures, requires higher computational resources, and often results in dehazed images with less realism compared to supervised learning methods.

In this paper, to address the issues of the lack of realism and the requirement for high computational resources in existing unsupervised learning methods, we propose an Unsupervised Image Dehazing Fusion Network (UIDF-Net). This network integrates unsupervised and supervised learning approaches for image dehazing, aiming to combine the advantages of both types of models to produce dehazed images that exhibit enhanced realism.

Our primary contributions are outlined as follows:

- We propose a novel Unsupervised Image Dehazing Fusion Network, UIDF-Net, to enhance the quality of dehazed images.
- We have designed a haze encoder named Mist-Encode, which integrates frequency domain processing with attention mechanisms to enhance the efficiency of image dehazing.
- We have designed a deep learning-based image perceptual fusion model that effectively enhances image quality.

The article is structured as follows. Section 2 elaborates on the haze degradation mechanisms and discusses three representative dehazing methods. Section 3 provides a detailed introduction to our proposed network, UIDF-Net. Section 4 conducts experiments and analysis to validate the effectiveness of the model. Section 5 summarizes the contributions of this study and discusses future work.

## 2. Preliminaries

In this section, the haze degradation mechanisms in image dehazing are discussed, and three representative dehazing methods are introduced. From these discussions, the experimental directions for this paper were identified. Within the UIDF-Net framework, the haze removal effectiveness metric called DCPI was proposed, leveraging the existing dark channel prior algorithm, as detailed in Section 4.1. Additionally, to address the lack of realism in unsupervised learning image restoration, the integration of haze-free images generated by the AOD-Net network as features with unsupervised learning dehazed images will be explored. The main approach is elaborated upon in Section 3.3. Furthermore, based on the unsupervised learning CycleGAN model, the haze encoder called Mist-Encode is introduced to enhance image dehazing efficiency.

### 2.1. Atmospheric Scattering Model

**Figure 1:**
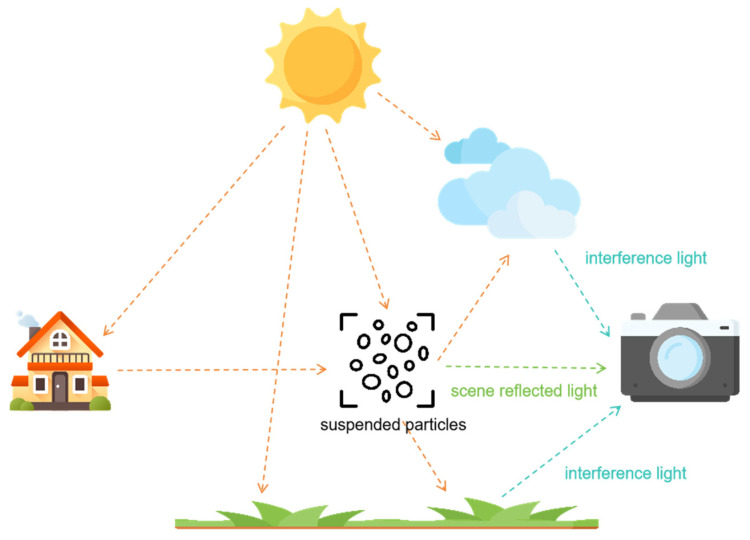
Imaging model of foggy conditions.

The scattering of particles in the atmosphere is the primary cause of haze [29]. Whether observed with the naked eye or captured in images, foggy conditions always present issues of reduced contrast and visibility. In 1999, Srinivasa G. Narasimhan and colleagues addressed the problem of low visibility in foggy conditions by establishing a mathematical model [30], explaining the imaging process of foggy images and the elements involved. The model suggests that in strong scattering media, there are two main reasons for the degradation of imaging results by detection systems: first, the absorption and scattering of suspended particles in the atmosphere affect the reflected light from objects, leading to a decrease in the energy of reflected light and, consequently, reducing brightness and contrast in the imaging results of the detection system; second, sunlight scattered by atmospheric scattering media forms background light, which typically has higher intensity than the light from the objects, resulting in unclear imaging results produced by the detection system. Figure 1 illustrates the imaging model of foggy conditions.

The diagram illustrates the imaging process of foggy conditions, showing that the light received by the detection system primarily originates from two sources: first, the light reflected from the target that reaches the detection system after attenuation by particles; second, the atmospheric light formed by the scattering of light sources (in this case, illumination) by particles. The mathematical model for foggy imaging is established based on this physical model.

(1) $$I\left(x,\lambda \right)=e^{-\beta \left(\lambda \right)d\left(x\right)}R\left(x,\lambda \right)+L_{\infty }\left(1-e^{-\beta \left(\lambda \right)d\left(x\right)}\right)=D\left(x,\lambda \right)+A\left(x,\lambda \right)$$

where $I\left(x,\lambda \right)$ is the foggy image obtained by the detection system, and $R\left(x,\lambda \right)$ represents the fog-free image that needs to be recovered. Parameter x represents the position of pixels in the image, and λ represents the wavelength of light. $L_{\infty }$ represents the atmospheric light value at infinity. t =$\;e^{-\beta \left(\lambda \right)d\left(x\right)}$ stands for the transport function, which is the proportion of light that reaches the detection system through particle attenuation.

### 2.2. Dark Channel Prior

The dark channel prior theory [10] is derived from observing a large number of outdoor haze-free images, revealing a statistical regularity: in most haze-free images that do not include sky regions, for each pixel in the image, at least one channel value is very small and close to zero. Using the dark channel prior theory, the minimum value across the three channels for each pixel in a hazy image is computed, forming the dark channel image of the hazy scene.

(2) $$J^{dark}\left(x\right)={min}_{y\in \Omega \left(x\right)}\{{min}_{c\in \left(R,G,B\right)}\left(J^{c}\left(y\right)\right)\}$$

where $J^{dark}\left(x\right)$ represents the pixel value of the dark channel of the image at the position of pixel x; $y\in \Omega \left(x\right)$ represents the rectangular neighborhood window in the center; C represents the channel of the image; and R, G, and B represent the red, green, and blue channels.

The dark channel prior dehazing algorithm proposes a practical estimation method as follows: first, identify the top 0.1% brightest pixels in the dark channel image. Then, among these pixels in the corresponding RGB channels, find the maximum value to determine the global atmospheric light value for the image. In computing the transmission map, the dark channel prior dehazing algorithm initially uses the dark channel image to estimate the haze density and provide an initial estimate of the transmission map. The specific calculation formula is as follows:

(3) $$\tilde{t}\left(x\right)=1-\omega {min}_{y\in \Omega \left(x\right)}\left({min}_{c\in \{R,G,B\}}\frac{I^{c}\left(y\right)}{A^{c}}\right)$$

where $\tilde{t}\left(x\right)$ is the estimated rough transmittance value, $A^{c}$ is the estimated atmospheric light value of the three channels of the image, and ω as 0.95 is generally taken. Therefore, the dark channel prior algorithm takes the above atmospheric scattering model as the theoretical model of fog imaging when it obtains fog images through the detection system and defogs them. By estimating transfer function $\tilde{t}\left(x\right)$ from foggy images and substituting the obtained parameters into the atmospheric scattering model, the target image R (x, λ) can be recovered.

### 2.3. AOD-Net Network Architecture

The AOD-Net algorithm aims to remove haze from images by learning the atmospheric light and transmission map, thus achieving the end-to-end restoration of clear images [18].

**Figure 2:**
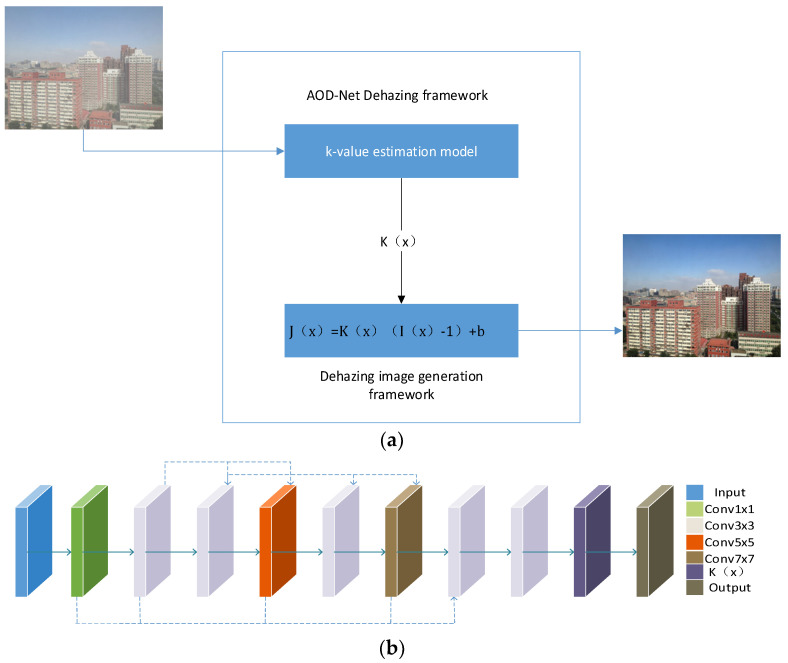
AOD-Net network architecture diagram. (**a**) The diagram of AOD-Net. (**b**) K-estimation module of AOD-Net.

The core idea of AOD-Net is to model the image dehazing process as a deep convolutional neural network that predicts the transmission map and atmospheric light from input hazy images. The transmission map represents the degree of light attenuation as it propagates through haze, while atmospheric light refers to the light generated by scattering from particles and molecules in the atmosphere. Specifically, the architecture of AOD-Net typically includes a sub-network for estimating the transmission map and another sub-network for estimating atmospheric light. These sub-networks learn the mapping from hazy images to transmission maps and atmospheric light, enabling the model to effectively infer values for transmission maps and atmospheric light and use them to generate haze-free images. AOD-Net can be trained end-to-end using a large amount of labeled data without prior knowledge. This allows the model to learn complex image dehazing processes and achieve good performance across various scenes and conditions. The network structure of AOD-Net is illustrated in Figure 2.

### 2.4. CycleGAN Network Architecture

**Figure 3:**
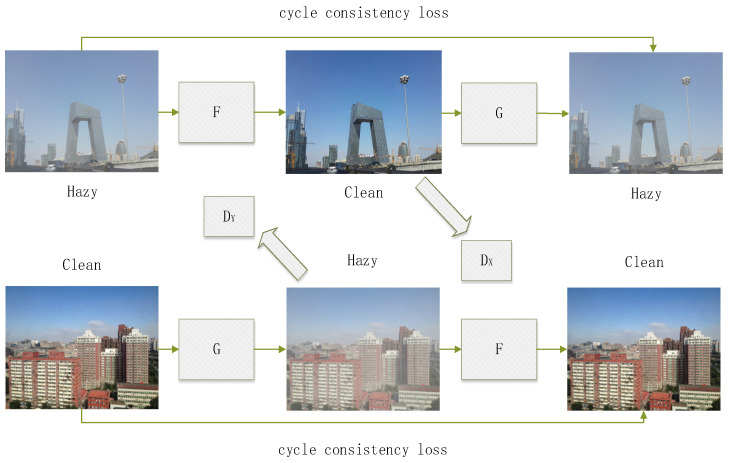
CycleGAN dehazing network architecture.

Generative Adversarial Networks possess powerful data generation capabilities and are widely applied in image generation, image style transfer, data augmentation, and other domains. However, due to the challenge of using GANs for specific image style transformations, Cycle-Consistent Generative Adversarial Networks [31] were introduced. During training, a CycleGAN requires only samples from domains X and Y without the need for a one-to-one correspondence between images in domain X and domain Y. This greatly alleviates the burden of dataset preparation. The trained model can transform images from domain Y style to domain X style, earning recognition in the field of style transfer [32,33,34]. Many researchers have begun applying CycleGANs to various practical tasks [35,36,37], such as converting grayscale images to color images or transforming images into nostalgic-style images. Image dehazing can also be viewed as an image style transfer problem, where the goal is to convert a blurry, hazy image into a clear, haze-free image. Figure 3 illustrates the principle of CycleGAN for dehazing.

## 3. Proposed Method

In this chapter, we mainly discuss the Unsupervised Image Dehazing Fusion Network (UIDF-Net), which consists of two main components. The first part involves obtaining dehazed images using an enhanced CycleGAN network and the AOD-Net dehazing method. The second part involves a perceptual fusion strategy using the MDL-IFM model, which combines the features of the obtained dehazed images to produce the final dehazed image.

### 3.1. UIDF-Net Network Structure

**Figure 4:**
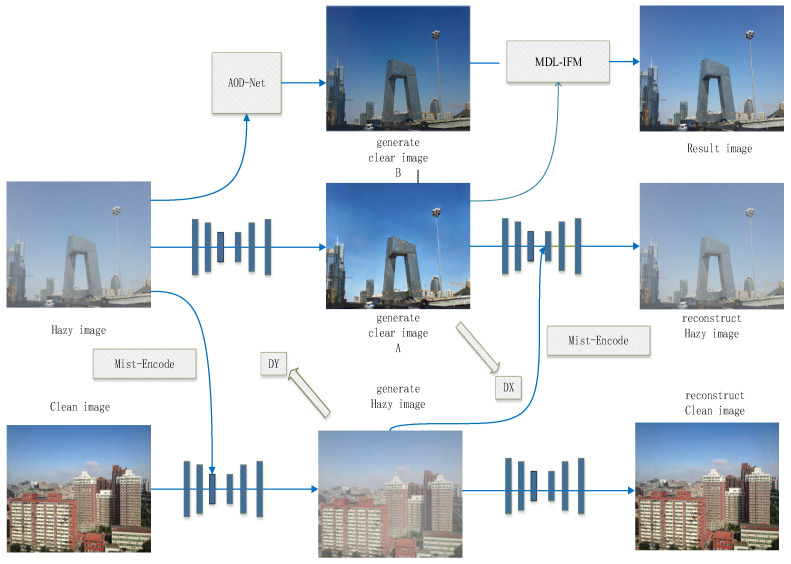
UIDF-Net network structure.

The network structure of the Unsupervised Image Dehazing and Fusion Network (UIDF-Net) is illustrated in Figure 4.

Image Dehazing Stage: Under the architecture based on CycleGAN [35], we introduced the Mist-Encode module. In this new CycleGAN framework, unpaired clear and hazy images (hazy image; clean image) are inputted into the network. The Mist-Encode module effectively separates haze information from the hazy images using multi-frequency representations, thereby generating clear image A. Simultaneously, the extracted haze information is added back into the clear images, transforming them into corresponding pseudo-hazy images (generate hazy image). Subsequently, through reverse reconstruction, we obtain reconstructed images (reconstruct hazy image; reconstruct clean image). Finally, adversarial learning is applied to refine these images, ensuring optimal dehazing effects. Additionally, the hazy images pass through the AOD-Net model to generate a new clear image, B.

Image Fusion Stage: In the previous stage, we obtained two dehazed images using the unsupervised CycleGAN model and the supervised AOD-Net model. In this stage, we propose the MDL-IFM module, which encodes the two dehazed images separately using content encoders. Ultimately, the encoded results are averaged to produce the corresponding clear image (Result image).

Next, we will detail the working principles of the Mist-Encode module and the MDL-IFM module.

### 3.2. Mist-Encode

**Figure 5:**
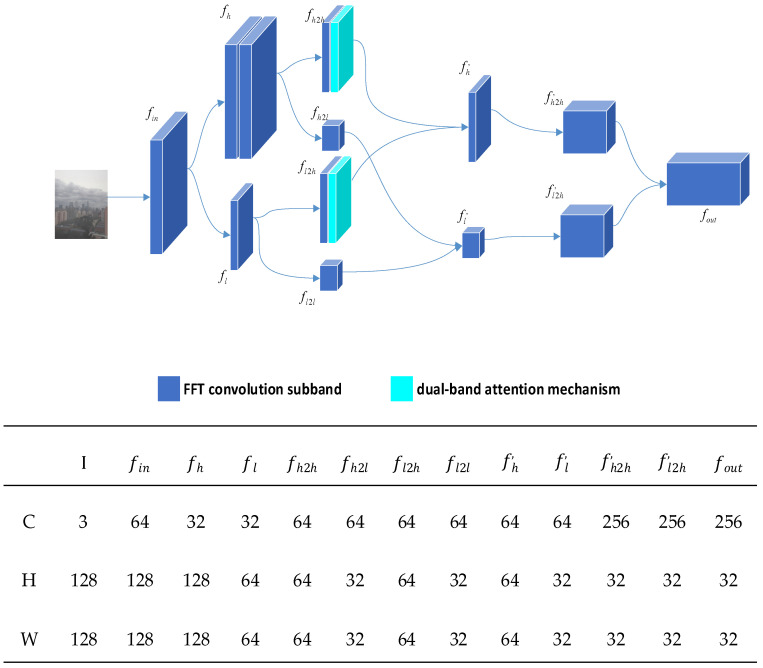
Mist-Encode structure.

In the first chapter, we elaborated on the issues of high computational resource requirements and lack of realism associated with unsupervised learning. Therefore, this section primarily proposes Mist-Encode to address the problem of high computational resource utilization. The implementation of Mist-Encode draws inspiration from the research findings of Li et al.’s USID-Net network, particularly the OctEncoder [20,37,38]. We further integrate FFTconvolution [39] and dual-band attention mechanisms [40,41] into its operations. After dividing the image into frequency bands, we calculate spatial and frequency-dimensional attention weights for the high-frequency feature maps using spatial attention and frequency attention mechanisms. This process yields weighted high-frequency feature maps. We demonstrated the effectiveness of the Mist-Encode module in subsequent ablation experiments. Figure 5 illustrates the network structure of the Mist-Encode model.

The Mist-Encode module first performs frequency domain convolution on the received hazy image, achieving a bifurcation of haze image information into high-frequency (fh) and low-frequency (fl) feature maps.

(4) $$f_{h}=FFTConv\left(f_{in}\right),f_{l}=FFTConv\left(f_{in}\right)$$

(5) $$f_{h2h}=FFTConv\left(f_{h}\right),f_{h2l}=FFTConv\left(f_{h}\right)$$

Perform dual-band attention operation on the high-frequency feature map after further downsampling.

(6) $$\alpha_{1}=frequency\_attention(f_{h2h})$$

(7) $$\alpha_{2}=frequency\_attention(f_{l2h})$$

(8) $$\beta_{1}=spatial\_attention(f_{h2h})$$

(9) $$\beta_{2}=spatial\_attention(f_{l2h})$$

The weighted result is as follows:

(10) $$f_{out}={\alpha_{1}*\beta_{1}f}_{h2h}+{\alpha_{2}*\beta_{2}f}_{l2h}$$

### 3.3. Perception Fusion Strategy MDL-IFM Model

**Figure 6:**
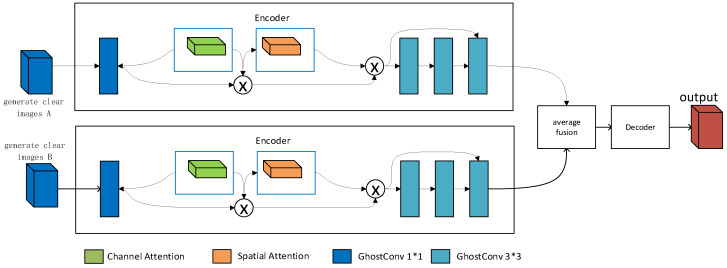
MDL-IFM network structure.

In Section 3.2, a haze encoder called Mist-Encode was designed to address the issue of high computational resource usage in unsupervised learning. In this section, the focus is on elaborating the perceptual fusion strategy MDL-IFM, Figure 6 shows the network structure of MDL-IFM. This model primarily aims to tackle another drawback of unsupervised learning, which is the lack of realism in the generated images. The key approach involves the hierarchical fusion of dehazed images obtained from unsupervised and supervised learning, aiming to maintain the perceptual realism achieved by supervised dehazing.

To implement this approach, an additional image fusion model is integrated after the dehazing model. To avoid compromising the computational efficiency addressed in Section 3.2, the fusion model must prioritize lightweight design. Therefore, we propose a lightweight MDL-IFM network structure. The network adopts an encoder–decoder paradigm. In the encoder, spatial attention mechanisms [41] and channel attention mechanisms [42] are employed to better represent image features. Feature encoding utilizes residual blocks [43]. The MDL-IFM network primarily encodes the generated images obtained in the first stage. Subsequently, the encoding results of the two images are averaged using an average fusion module to fuse dehazed images from unsupervised and supervised learning. Finally, the decoder is used to reconstruct the final image.

In addition, deep convolutional neural networks typically employ large numbers of convolution layers, resulting in high computational costs. To achieve lightweight model design goals, GhostConv [44] replaces traditional convolution operations in the MDL-IFM network structure. Compared to regular convolution, GhostConv can significantly reduce model parameters and computational workload while maintaining model accuracy. Specifically, GhostConv divides a conventional convolution kernel into two parts: a smaller primary convolution kernel and a larger secondary convolution kernel. The primary kernel is used for feature extraction, while the secondary kernel increases channel capacity. During each forward pass, GhostConv computes only with the primary kernel, and the secondary kernel processes each channel once, concatenating the results. This approach enables GhostConv to reduce model parameters effectively while preserving model efficiency and performance.

## 4. Experiment

### 4.1. Experimental Details

Dataset Description: In practical engineering, haze primarily exists outdoors. Therefore, this experiment focuses on investigating the model’s performance in dehazing outdoor images. Two different outdoor datasets were used for comparison in this study, while indoor haze images were disregarded. Dataset A: 5000 pairs of high-resolution images and artificially synthesized haze images were selected from the RESIDE datasets [45]. The dataset was split into training, validation, and test sets in a ratio of 7:2:1. Dataset B: This study collected 500 haze images of mountainous transmission lines taken from real cameras. The dataset is referred to as TLD.

Training configuration: To achieve effective model training and evaluation, the UIDF-Net network model was trained on the PyTorch deep learning platform using an NVIDIA GeForce RTX 3060 graphics card. During training, the model utilized an input image size of 128 × 128 pixels and a batch size of 2, and the learning rate for the Adam optimizer was set to 0.0001, with exponential decay after 10,000 iterations.

Comparison and Metrics: We will compare the proposed UIDF-Net model with widely recognized algorithms. These include prior-knowledge-based methods, such as DCP and CAP, supervised learning approaches, like DehazeNet and AOD-Net, and unsupervised learning techniques, including CycleDehaze, USID, and RefineNet. To assess the dehazing performance of each model, we will use peak signal-to-noise ratio (PSNR) and structural similarity index (SSIM) as evaluation metrics. However, Dataset B, comprising self-collected transmission line images, lacks high-definition, noise-free original images, making these metrics unsuitable. Therefore, the experiment requires a metric that does not rely on high-definition images as references to evaluate the model’s dehazing performance in complex environments such as actual transmission line scenarios. In the field of dehazing, the dark channel prior offers valuable insights: the dark channel values in the original image are typically minimally affected by haze, whereas the dehazed image should exhibit higher dark channel values. Thus, by analyzing changes in the dark channel prior, it is possible to effectively assess and compare the performance of different dehazing algorithms, as well as their capability to restore image details. Inspired by this theory, this experiment aims to quantify dehazing effectiveness by measuring changes in the dark channel prior values between original and dehazed images. The specific steps are outlined as follows:

Given an image I with three channels IR、IG、IB, for each channel $c\in \left(R,G,B\right)$, we compute its result after applying an erosion operation $I_{c}^{\prime }$. Then, using Formula (2), obtain the minimum value across the three channels as the dark channel J.

(11) $$I_{c}^{\prime }=erosion\left(I_{c},kernel\right)$$

where $I_{c}^{\prime }$ represents the pixel value of image $I_{c}$ at position (x,y) after applying the erosion operation, and kernel represents the structuring element used for the erosion operation. After obtaining the dark channel prior values before and after dehazing, calculate their means, mean_before and mean_after. Then, compute the improvement in dehazing effectiveness by subtracting the mean values of the dark channel prior before and after dehazing. We name this improvement measure DCPI (Dark Channel Prior Improvement).

(12) $$\mathrm{mean}\_\mathrm{before}=\frac{1}{N}\sum_{\left(x,y\right)}J_{\mathrm{before}}\left(x,y\right)$$

(13) $$\mathrm{mean}\_\mathrm{after}=\frac{1}{N}\sum_{\left(x,y\right)}J_{\mathrm{after}}\left(x,y\right)$$

DCPI = mean_before − mean_after (14)

Here, N represents the total number of pixels in the image.

### 4.2. Comparison of Dehazing Effects with Different Algorithms

It is worth noting that, to ensure the effectiveness of the metrics, the parameter settings of the comparison algorithms were all referenced from relevant literature, and uniform experiments were conducted on the aforementioned equipment to quantitatively evaluate the performance of different algorithms in dehazing. Table 1 shows the dehazing metrics of various algorithms under two different datasets.

**Figure 7:**
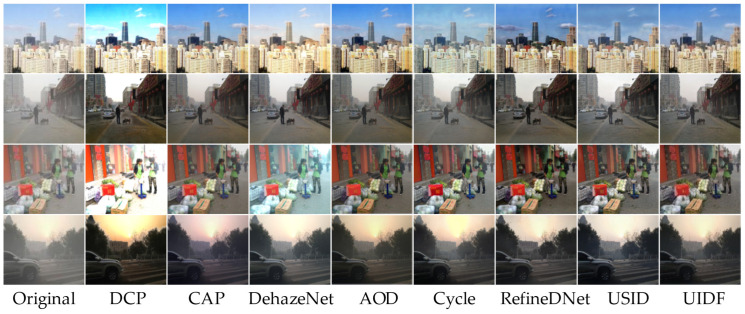
Comparison of algorithm performance on Dataset A.

Dehazing Results on Dataset A: In experimental testing, the supervised learning model AOD-Net demonstrated significant advantages in metrics, attributed to Dataset A being trained with paired blurry–clear images, thereby fully leveraging its feature extraction capabilities. Our proposed algorithm also achieved promising results. Figure 7 illustrates the dehazing results of the comparative algorithms and UIDF-Net algorithm.

**Figure 8:**
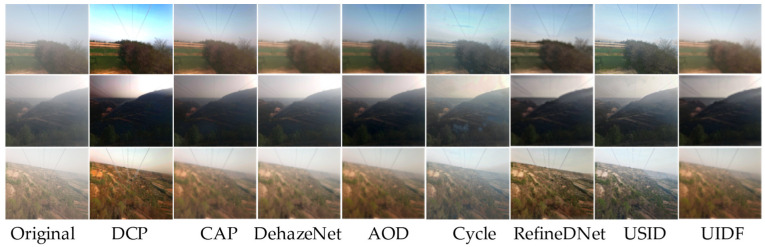
Comparison of algorithm performance on Dataset B.

Dehazing Results on Dataset B: Due to the nature of transmission line images lacking high-definition, noise-free originals, the training of AOD-Net still utilized the training weights from Dataset A. In experimental testing, unsupervised learning showed significant advantages in performance, and the UIDF-Net algorithm also demonstrated clear benefits. This is attributed to the more complex environment where supervised learning lacks corresponding paired datasets, resulting in poor generalization. In contrast, unsupervised learning can mitigate such issues. Figure 8 illustrates the dehazing results of the comparative algorithms and the UIDF-Net algorithm.

Additionally, the complexity of the proposed model was evaluated under the same experimental conditions. UIDF-Net was compared with other benchmark algorithms in terms of runtime and trainable model parameters, as quantified in Table 2. AOD-Net and USID-Net demonstrated excellent performance in both model parameter count and runtime. Based on this, UIDF-Net, primarily informed by these two models, also achieved favorable results.

### 4.3. Component Removal Study

To investigate the effects of each module in the proposed UIDF-Net on image dehazing, we conducted four corresponding experiments. Among them, M1 represents the dehazed images using the USID-Net. M2 adds the Mist-Encode module on top of M1, and M3 adds the MDL-IFM module on top of M1. M4 integrates both the Mist-Encode and MDL-IFM modules into the UIDF-Net network.

**Figure 9:**
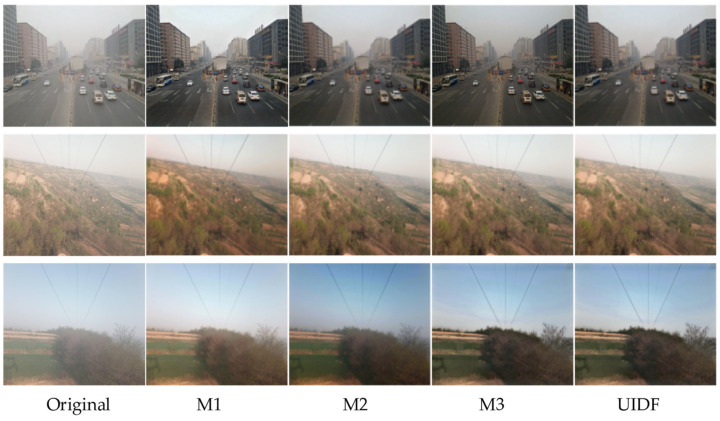
Dehazing effects at each stage.

The dehazing performance of each model on two datasets is illustrated in Figure 9. Using M1 as the baseline for comparison, we observed that M2 achieved an approximately 25% increase in speed during network training compared to M1. The dehazed images from M3 exhibited richer and more realistic details. M4 combined M2 and M3 to address two major drawbacks of unsupervised learning.

Finally, the de-fog metrics for each model on the two data sets are shown in Table 3. It can be observed that M2 focuses on improving speed, thus it does not outperform M3 in dehazing metrics. M3 shows an improvement in the metrics. Ultimately, the UIDF-Net network demonstrates good performance in dehazing.

## 5. Conclusions and Future Work

In this paper, we propose a UIDF-Net network to address the issues of high computational cost and lack of realism in single-image dehazing using Generative Adversarial Networks (GANs) in complex real-world environments. Within the network, we introduce the Mist-Encode module to swiftly decompose the frequency-domain information of hazy images and establish feature connections between the dehazing and reconstruction branches of GANs to enhance dehazing speed, resulting in a 25% increase in training efficiency. Furthermore, the MDL-IFM module is employed for haze image fusion, improving the quality of recovered images. Our proposed model achieves a PSNR score of 22.58 and a DCPI score of 72.36, demonstrating superior performance compared to existing algorithms. In future work, we aim to integrate scene depth and lighting features into the fusion process to achieve multi-modal fusion and thereby accomplish more comprehensive image dehazing tasks.

## Figures and Tables

**Table 1:** Comparative algorithm performance metrics (top three rankings identified in red, green, and blue order).

Type	Methods	Dataset A			Dataset B
		PSNR	SSIM	DCPI	DCPI
Prior	DCP	18.20	0.81	-	-
	CAP	22.45	0.90	48.53	45.41
Supervised	DehazeNet	19.52	0.82	24.81	29.71
	AOD-Net	21.01	0.89	64.81	53.56
Unsupervised	Cycle-Dehaze	18.87	0.83	53.64	48.26
	RefineDNet	20.79	0.88	64.73	55.69
	USID-Net	21.40	0.81	51.15	44.12
	Ours	22.58	0.87	72.36	51.85

**Table 2:** Model complexity assessment (top three rankings identified in red, green, and blue order).

Type	Methods	Parameters (M)	Runtime (s)
Prior	DCP	-	0.1837
	CAP	-	0.9235
Supervised	DehazeNet	0.008	1.5269
	AOD-Net	0.002	0.0038
Unsupervised	Cycle-Dehaze	11.380	-
	RefineDNet	64.375	0.5626
	USID-Net	3.820	0.0189
	Ours	3.996	0.0275

**Table 3:** Dehazing metrics at each stage.

Methods	Dataset A			Dataset B
	PSNR	SSIM	DCPI	DCPI
M1	21.40	0.81	51.15	44.12
M2	21.42	0.80	51.25	46.26
M3	22.40	0.86	66.54	49.38
UIDF	22.58	0.87	72.36	51.85

## Data Availability

Data sharing applicable.
